# Preschool Cyber Security Management System Based on Intelligent Agents

**DOI:** 10.1155/2022/1992429

**Published:** 2022-10-07

**Authors:** Jing Song

**Affiliations:** Zhengzhou Preschool Education College, Zhengzhou 450000, China

## Abstract

As information and communication technologies create an ever-increasing complexity in interconnected systems and devices, cybersecurity and privacy issues are constantly at the fore, highlighting the need to strengthen the protection and resilience of these systems against the ever-evolving threats of modern cyberspace. This particular work, taking into account that preschool children now have significant needs to ensure their digital identity and, in general, their protection from their contacts with the internet, aspires to provide an understandable and practical guide to strengthen the security of information systems and information from both public and private school agencies. Specifically, a preschool cybersecurity management system based on intelligent agents is proposed. Using sophisticated, intelligent techniques, it aims to improve the ability of preschools to resist modern threats adequately, respond to cyber-attack incidents with the least possible impact, and protect their critical systems, services offered, and the personal data they hold and process. The system intends to link and control distributed systems that currently exist, as well as to solve issues that are beyond the knowledge and skills of a single agent. This novel research idea has never been offered in the relevant literature, and we think it has the potential to advance the state of the art in cybersecurity significantly.

## 1. Introduction

Today's typical structure of the information systems of an educational institution such as preschools has reached an exceptionally high degree of complexity [1]. Their essential characteristics include at least a central building infrastructure with servers that have public IP (web, mail, DNS, etc.) and with various internal networks that host employees' office computers or other infrastructures used in education [2]. Sometimes, employees bring their own portable devices (laptops, tablets, and smartphones) connected to the carrier's network to the workplace and their mobile storage media (USB, external hard drives, etc.) [3].

The remote offices of the same organization in other regions with their own corresponding internal network infrastructure should be added to the infrastructures in question. The operator's applications assist the specific computing systems, usually web, hosted in the data centers of one or more cloud service providers [4]. Recently, due to the pandemic, the institution's employees, as well as the teachers, work from home (teleworking), connect remotely to the institution's internal network and handle critical data using a home network and computers that have not been tested and certified in terms of their safety [5, 6]. Also, third-party providers and suppliers who have undertaken the development of applications and the technical support of the organization's systems connect remotely to its internal network through their infrastructure or have assigned the work to their subcontractor [7].

As is logical, the sensitive data of educational institutions, especially those related to preschool children, should be supported by information systems that are at least aligned with in-depth defense architecture. In this model, security measures and mechanisms are applied in successive layers across the entire scope of an operator's network and data to protect them from threats [8]. Each layer individually does not deal with all threats, while they deal with a wide variety of offensive techniques. If a threat manages to bypass a layer, it must deal with the defense mechanisms of the next layer.

**Figure 1:**
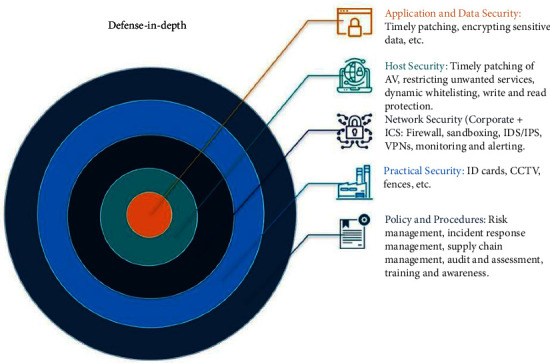
Defense in depth (https://modernciso.com/).

An effective defense-in-depth strategy includes mechanisms at the purely technical level, as well as organizational or administrative measures, such as policies and procedures (risk analysis, user training, personal data management, etc.), access restrictions (least privilege, need-to-know, etc.), network security (network segmentation, firewalls, intrusion detection systems, VPNs, etc.), device protection (antivirus, application whitelisting, etc.), and application and data protection (patching, data backup, encryption, etc.) [9].  Figure 1 graphically illustrates an example of the sequential layering of defense-in-depth architecture.

We observe a broad and difficult-to-control dispersion in an operator's data processing, storage, and circulation. At the same time, the traditional network perimeter is no longer demarcated. The above is happening in an interconnected world becoming increasingly vulnerable to malicious activity as connectivity, device richness, distributed applications and services, and complexity in cloud and multicloud environments increase [4, 10, 11]. It is clear that such complexity dramatically increases the security requirements to protect the carrier's critical data from leakage, intentional alteration, or even disruption of availability. Various architecture models have been proposed for the effective defense against constantly evolving threats, which are found in the relevant research literature. But this novel research idea has never been offered in the relevant literature, and we think it has the potential to advance the state of the art in cyber security significantly.

The rest of this paper is structured as follows: Section 2 presents the relevant research studies.  Section 3 is allocated to the presentation of the proposed system.  Section 4 presents the protocol of the agreement, and finally, section 5 concludes the research.

## 2. Relevant Research Studies

The literature on the utilization of intelligent agents keeps expanding and covers not only the security aspect of research but also the safety or other perspectives.

Kasereka et al. [12] suggested in 2018 a smart agent-based model to model and simulate the removal of individuals from a burning building. Their concept has been founded on four criteria that allow for her realistic evaluation. In a simulated case study conducted in a building with the general layout of a supermarket, it was determined that the presence of multiple individuals to be removed, the consideration of fire propagation speed, and other aspects significantly impacted the model. This concept is sufficiently broad to be applied in multiple kinds of business buildings without significant modification. We seek to integrate these perspectives into the framework by incorporating a fuzzy approach into the system.

Kotenko et al. [13] presented a strategy for implementing intelligent agents for internet and vulnerability risk assessments in cyber-physical systems. The proposed method has a much smaller sliding window size than the sliding window technique with the same accuracy of assessing traffic characteristics, runs in real-time, and uses fuzzy logical inference to regulate parameters. The experimental evaluation supports the method's fast speed and appropriate precision for analyzing network data. Simultaneously with time, it offers evaluation accuracy equivalent to that of established algorithms. Methods of dynamic supervision of intelligent agents in cyber-physical security systems are the subject of more study.

Kushal et al. [14] developed a two-pronged technique to minimize the consequences of an unusual false data injection attempt, in which an attacker uses batteries to actively lower load curtailment to introduce updates to the central control system. An intelligent agent system examines directives from the primary energy administration system. A bilevel technique is constructed to describe the relationship between the cell and the hacked shipboard power system to discover symptoms of fraudulent data. They developed a heuristic defensive parameter to enhance the detection of tainted instructions. A danger assessment model is used to assess the advantages of the proposed strategy. The findings of the case studies demonstrate that a mixture of an autonomous battery and a heuristic strategy helps reduce the impacts of a cyberattack.

Manbachi and Ordonez [15] suggested an advanced agent-based method for the power management of AC-DC microgrids on isolated islands. This strategy supplied islanded ac-dc microgrids with three major operations interacting at each functioning time interval to improve system performance, efficiency, and dependability. Between the agents, bidirectional communication enabled data gathering and command flow control. They employed an innovative multiobjective particle optimization engine to successfully address each agent's issue for the goal of optimization. A microgrid with various ac-dc producing assets and loads was analyzed to evaluate the accuracy and practical efficiency of the proposed solution.

Biregani and Fotohi [16] presented a countermeasure against malicious UAV assaults. In the first stage, numerous criteria and principles were implemented to identify malicious UAVs. In phase two, a mobile agent was employed to destroy malicious UAVs by alerting regular neighbor UAVs not to listen to the data produced by malicious UAVs. The conjunction of these two steps resulted in secure interactions between the UAVs, allowing packets to exchange data safely. The simulation results demonstrated that the suggested strategy is superior to previous approaches. To detect hostile UAVs in future work, they propose integrating two or more innovative and optimum algorithms, such as earthworm efficiency algorithm, moth search method, monarch butterfly optimum, and elephant herding utilization.

Alhayani et al. [17] investigated the efficacy of artificial intelligence solutions against cyber security threats. They mostly used quantitative research methods and collected primary data from IT sector personnel. Using confirmatory pattern evaluation, discriminant validity, fundamental model analysis, and hypothesis testing, they determined that intelligence agents, and artificial neural networks strongly influenced synthetic intelligence approaches. The development of technology has expanded data storage, necessitating better data security.

## 3. Proposed System

Education agencies increasingly depend on information and communication technologies to carry out their day-to-day operations and mission [1, 2]. These technologies are subject to threats, which exploit known and unknown system vulnerabilities with possible severe effects on business operations, persons, infrastructures, and the safeguarding of sensitive personal data, due to the violation of the confidentiality, integrity, and availability of the information that these systems process, store, or transmit. Threats to IT include cyber-attacks, human errors, and structural failures [8, 18, 19].

For the above reasons, it is imperative for educational institutions, especially preschool education institutions, to realize their responsibility and establish a comprehensive organizational approach to risk management related to the operation and use of information systems.

A vital component of a risk management framework is risk assessment, which consists of the following series of actions [3]:

1. The sources of threats related to the operator are identified (malicious groups, competitors, natural threats, errors, etc.)
2. Actions/events (threat events) that could occur from the above sources (cyber-attacks, hardware failures, etc.) are identified
3. The vulnerabilities of the organization that a source could exploit through specific actions/events are identified
4. The probability that the identified sources will initiate specific actions and the probability of successful realization of the events are estimated
5. The adverse effects (on the operations and systems of the entity, on persons, or other organizations) if the actions/events take place are assessed
6. The risk to the operator's security is determined as a combination of the probability of the events and the adverse effects if the events occur

Based on the calculated risk, the operator should choose the corresponding protection measures to address the risks adequately. Also, the organization should develop a security policy [20, 21], which will define at a high level the security goals and the organization's approach to achieving them while referring to more specific thematic policies and procedures that will specify the implementation and application of the selected protection measures.

Risk management is always the starting point for a practical approach to cyber security. Thus, the agencies must establish an information security management system, which is as follows:

1. Will be implemented by implementing technical and organizational security measures that will be based on risk management
2. Will have the full financial and organizational support of the administrative leadership
3. Will be inspected and renewed at regular intervals and
4. Will shape a cyber security culture for all involved (from senior management to all involved staff)

An appropriate organizational structure with responsibility for the security of information systems should be created for an information security management system to be implemented effectively [22]. In this structure, it should

1. Define the appropriate roles and responsibilities
2. Be adequately staffed with persons possessing technical and legal expertise in cyber security issues and
3. Allocate the required resources for the implementation of the goals set for cyber security

To automate the above functions and make them independent of human experience and knowledge, this work is proposed to develop a preschool cyber security management system based on intelligent agents [13]. Intelligent agents are modern artificial Intelligence systems that can be used selectively and combined with knowledge representation and problem-solving methods with advanced modern computing technologies [17]. Intelligent agents are computational systems that operate in a complex environment and perceive and act autonomously. In this way, they achieve a set of goals and perform tasks for which they are designed. Intelligent agents continuously perform three functions: they perceive the dynamic conditions of the environment, they act on the background to change it, and they reason to interpret what they perceive, solve problems, and draw conclusions to determine their actions [23].

The proposed system is a multiagent intelligent system that consists of a set of agents that act together to solve the given problem of cyber security management [24, 25]. The system aims to interconnect and operate already existing systems that are distributed, as well as to solve problems that are beyond the capabilities and knowledge of a single agent. Multiagent systems are a vital domain of distributed AI from a loosely considered view of agents, where relevant knowledge is distributed across discrete sources, such as existing experience in individual agent systems.

The proposed multiagent network is a set of agents with dynamic behavior interacting to achieve a common goal. The system in question includes any type of network or system consisting of spatially distributed autonomous devices that collectively record conditions and communicate with each other with wireless or wired devices, exchanging information to achieve an accurate estimate for the desired variable [15]. The system proposes to connect and control existing dispersed systems, as well as to solve problems that are beyond the knowledge and talents of a single person.

An essential element of networked systems, which separates them from the systems that have traditionally been considered in systems theory, is the existence of the network and its effect on the whole system's behavior. The geometry of the network imposes constraints on its behavior, as well as the interactions between agents, described by the graph theory translation of agents as nodes and interactions as branches of a graph representing the network. In such a graph, the existence of a branch indicates that the connected nodes interact with each other.

The agreement is one of the fundamental problems of multiagent coordination, in which a collection of agents must agree on a common state value [24, 26]. In this work, the dynamics of the agreement protocol for undirected static networks are studied to implement the multiagent network of cyber security management.

## 4. The Protocol of the Agreement

Let there be a multiagent network in which the agents must perform some measurement [27]. Although each measurement made by the individual agents will differ due to its location, it is necessary to reach an agreement on a specific value, which will be achieved by sharing the agents' information. For this purpose, the agents need some communication protocol that will act on the network and allow it to achieve the agreement. The agreement protocol includes *n* dynamic units, denoted as 1,2, ..., *n* and are connected by a communication bus between them. Let the state of unit *i* be *x*_*i*_ ∈ *R*. Then, the protocol has the form [28]:

(1) $$\begin{array}{c} {\dot{x}}_{i}=-\underset{j\in N\left(i\right)}{\sum }\left(x_{i}\left(t\right)-x_{j}\left(t\right)\right),i=1,\cdots ,n\text{,} \end{array}$$

where *N*(*i*) is the set of neighbors of *i* in the network [29]. The overall network then has momentum

(2) $$\begin{array}{c} \dot{x}\left(t\right)=-L\left(G\right)x\left(t\right)\text{,} \end{array}$$

where the positive semidefinite matrix *L*(*G*) is the Laplacian of the interaction network of agents *G* and *x*(*t*)=(*x*_1_(*t*),…,*x*_*n*_(*t*))^*T*^ ∈ *ℝ*^*n*^. The above equation will be referred to as agreement dynamics. With this protocol in action, node potentials are drawn toward the states of neighboring nodes [30]. The value they finally arrive at, i.e., the agreement state, is defined by the agreement set *A*⊆*ℝ*^*n*^ which is the subset span  {1} which is

(3) $$\begin{array}{c} A=\left\{\left.x\in R^{n}\right|x_{i}=x_{j},\forall i,j\right\}\text{,} \end{array}$$

To clarify the mechanism by which dynamic agreement in an undirected graph drives network nodes to the agreement state, one should consider the eigenvalues of the Laplacian of a connected and undirected graph, which take the form [31]

(4) $$\begin{array}{c} 0=\lambda_{0}\left(G\right)\leq \lambda_{1}\left(G\right)\leq \ldots \leq \lambda_{n-1}\left(G\right)\text{,} \end{array}$$

where 1, the vector with all elements equal to unity, is the eigenvector corresponding to the zero eigenvalue *λ*_0_(*G*). Recall that the Laplacian is symmetric and *L*(*G*)1=0 for undirected G. Let *U*=[*u*_0_*u*_1_ … *u*_*n*−1_] be the matrix consisting of normalized and mutually orthogonal eigenvectors of *L*(*G*) which are assigned to the ordered eigenvalues. In addition, let [32, 33]

(5) $$\begin{array}{c} \Lambda \left(G\right)=Diag\left({\left[\lambda_{0}\left(G\right),\ldots ,\lambda_{n-1}\left(G\right)\right]}^{T}\right)\text{.} \end{array}$$

Applying the spectral theorem to the Laplacian, it yields

(6) $$\begin{array}{c} e^{-L\left(G\right)t}=e^{-\left(U\Lambda \left(G\right)U^{T}\right)t}=Ue^{-\Lambda \left(G\right)t}U^{T}\\ =e^{-\lambda_{0}\left(G\right)t_{0}}u_{0}^{T}+e^{-\lambda_{1}\left(G\right)t_{1}}u_{1}u_{1}^{T}+\ldots +e^{-\lambda_{n-1}\left(G\right)t}u_{n-1}u_{n-1}^{T}\text{.} \end{array}$$

Therefore, the solution of $\dot{x}\left(t\right)\in F\left[X\right]\left(x\left(t\right)\right)$, with initial value *x*(0)=*x*_0_ is

(7) $$\begin{array}{c} x\left(t\right)=e^{-L\left(G\right)t}x_{0}\text{,} \end{array}$$

which can be decomposed along each eigen-axis as [34]:

(8) $$\begin{array}{c} x\left(t\right)=e^{-\lambda_{0}\left(G\right)t}\left(u_{0}^{T}x_{0}\right)u_{0}+e^{-\lambda_{1}\left(G\right)t}\left(u_{1}^{T}x_{0}\right)u_{1}+\ldots +e^{-\lambda_{n}\left(G\right)t}\left(u_{n-1}^{T}x_{0}\right)u_{n-1}\text{.} \end{array}$$

The problem to be solved is to achieve agreement in a multiagent network with unknown disturbances for a stable network topology. Agents follow simple integrator dynamics. With blocked and continuous second-order derivatives, perturbations are considered blocked. The undirected network description graph is connected. The following solution employs discontinuous systems theory and distributed continuous control [28, 35].

The input of the individual agents is first filtered using the variables *T* and *β* as well as the sign of the error *ξ*. Then, using the filtered information, the final one is generated, which has an additional summation term using *ξ*. This control is fully distributed, that is, individual agents operate autonomously using information only from their neighbors. It is shown to asymptotically achieve agreement on a stable graph topology [36]. In practice, achieving convergence means that the input learns the perturbation and copes with it.

Specifically, the agent network topology is described using an undirected, connected graph where *G*=(*V*, *E*), where*V*={*u*_1_, *u*_2_,…, *u*_*n*_} is the set of its nodes and *E*⊆*V* × *V* all its branches [37, 38]. The set of neighbors of agent *i* is *N*(*i*)={*u*_*j*_ ∈ *V*|*u*_*i*_*u*_*j*_ ∈ *E*}.

The dynamics of the agents are given by the equation [39]:

(9) $$\begin{array}{c} {\dot{x}}_{i}=u_{i}+d_{i},i\in V\text{,} \end{array}$$

where ${\dot{x}}_{i}$ are the one-dimensional state variables of the agents, and *d*_*i*_ are unknown blocked perturbations with continuous blocked derivatives up to the second degree and

(10) $$\begin{array}{c} u_{i}=-K_{p}\xi_{i}+u_{fi},i\in V\text{,} \end{array}$$

*u*_*fi*_ is the filtered input

(11) $$\begin{array}{c} T{\dot{u}}_{fi}+u_{fi}=-\beta sgn\left(\xi_{i}\right),i\in V\text{,} \end{array}$$

where *sgn* is the sign function and *T*, *β*, *Kp* are control gains. *ξ*_*i*_ contain the information of agent *i* about its neighbors and are given by the equation [40]

(12) $$\begin{array}{c} \xi_{i}=\underset{j\in N\left(i\right)}{\sum }\alpha_{ij}\left(x_{i}-x_{j}\right),i\in V\text{,} \end{array}$$

The problem is to show that agreement is reached, i.e., that *x*_*i*_ − *x*_*j*_⟶0, ∀*i*, *j* ∈ *V* for fixed graph topology [41].

For the existence of solutions of the system, a Lyapunov function will be used as follows [24, 31, 42, 43]:

(13) $$\begin{array}{c} V\left(x_{ag},t\right)=\frac{1}{2}{\left(\dot{x}+\frac{1}{T}x\right)}^{T}L\left(\dot{x}+\frac{1}{T}x\right)+\frac{\beta }{T}\underset{i\in V}{\sum }\left|\xi_{i}\right|-\underset{i\in V}{\sum }\;\left({\dot{d}}_{i}+\frac{1}{T}d_{i}\right)\xi_{i}\text{,} \end{array}$$

where $x_{ag}=\left(x,\dot{x},d,\dot{d}\right)$ the augmented state vector with $x,\dot{x},d,\dot{d}\in {\mathbb{R}}^{n}\text{ and }ℒ$ the Laplacian of the network graph. All terms of the function are continuously differentiable and therefore normal, except for the term containing the absolute value of *ξ*_*i*_. By the original hypothesis theorem, this term is also normal, so the Lyapunov function is also normal [44–46].

Using the eigenvalue decomposition of the Laplacian and since the Laplacian has a unique zero eigenvalue, we get [27, 45]

(14) $$\begin{array}{c} L=\left[\begin{array}{cc} U & \frac{1_{n}}{\sqrt{n}} \end{array}\right]\left[\begin{array}{cc} \sum & 0\\ 0 & 0 \end{array}\right]\left[\begin{array}{c} U^{T}\\ \frac{1_{n}^{T}}{\sqrt{n}} \end{array}\right]\text{,} \end{array}$$

where *U* ∈ *ℝ*^*n*×(*n* − 1)^ with property *U*^*T*^*U*=*𝕀*_(*n* − 1)×(*n* − 1)_ and Σ ∈ *ℝ*^(*n* − 1)×(*n* − 1)^ has in positions (*i*, *i*), *i*=1,…, *n* − 1 the values $\sigma_{i}\left(L\right)=\sqrt{\lambda_{i}\left(L^{T}L\right)}\text{.}$

In the form presented, the matrix Σ has all nonzero eigenvalues of the Laplacian on its diagonal since the unique zero eigenvalue of the Laplacian has been removed. So, the Laplacian is written in the form [47, 48]

(15) $$\begin{array}{c} L=U\Sigma U^{T}=U\Sigma \Sigma^{-1}\Sigma U^{T}=U\Sigma U^{T}U\Sigma^{-1}U^{T}U\Sigma U^{T}=LU\Sigma^{-1}U^{T}L\text{.} \end{array}$$

Since the Laplacian has an eigenvector of 1, it also holds that

(16) $$\begin{array}{c} L=L\left(U\Sigma^{-1}U^{T}+r\frac{1^{T}}{N}\right)L\text{,} \end{array}$$

where *r* is a positive constant, which is chosen so that *λ*_1_(*ℒ*) < *r* < *λ*_max_(*ℒ*). If set

(17) $$\begin{array}{c} L_{0}=\left[\begin{array}{cc} U & \frac{1_{n}}{\sqrt{n}} \end{array}\right]\left[\begin{array}{cc} \sum^{-1} & 0\\ 0 & r \end{array}\right]\left[\begin{array}{c} U^{T}\\ \frac{1_{n}^{T}}{\sqrt{n}} \end{array}\right]=U\sum^{-1}U^{T}+r\frac{11^{T}}{N}\text{.} \end{array}$$

The request is satisfied.

Therefore, from the property *ξ*=*ℒx*, the function *V*_0_ can be written in the form [49, 50]

(18) $$\begin{array}{c} V_{0}=\frac{1}{2}{\left(\dot{\xi }+\frac{1}{T}\xi \right)}^{T}L_{0}\left(\dot{\xi }+\frac{1}{T}\xi \right)\text{.} \end{array}$$

So the function *V*_0_ is [49, 51] continuous on its set of values as a quadratic function and so holds

(19) $$\begin{array}{c} \frac{d}{dt}\left({\dot{x}}_{i}+\frac{1}{T}x_{i}\right)={\dot{d}}_{i}+\frac{1}{T}d_{i}-K_{p}\left({\dot{\xi }}_{i}+\frac{1}{T}\xi_{i}\right)+\frac{1}{T}\beta sgn\;\left(\xi_{i}\right),\forall i\in V\text{,} \end{array}$$

and so

(20) $$\begin{array}{c} \frac{dV_{0}}{dt}=-K_{p}\underset{i\in V}{\sum }{\left({\dot{\xi }}_{i}+\frac{1}{T}\xi_{i}\right)}^{2}+\underset{i\in V}{\sum }\left({\dot{d}}_{i}+\frac{1}{T}d_{i}\right)\left({\dot{\xi }}_{i}+\frac{1}{T}\xi_{i}\right)\\ -\underset{i\in V}{\sum }\frac{\beta }{T}sgn\left(\xi_{i}\right)\left({\dot{\xi }}_{i}+\frac{1}{T}\xi_{i}\right)\text{.} \end{array}$$

Therefore *V*_0_ is blocked and so

(21) $$\begin{array}{c} V_{0}=\frac{1}{2}{\left(\dot{x}+\frac{1}{T}x\right)}^{T}L\left(\dot{x}+\frac{1}{T}x\right)\geq \frac{\lambda_{\min}\left(L_{0}\right)}{2}{\left\|\dot{\xi }+\frac{1}{T}\xi \right\|}^{2}\text{.} \end{array}$$

So since *V*_0_ is closed and locally Lipschitz, it is Lipschitz continuous on the set of values of *x*_*ag*_.

So the Lyapunov function is [52, 53]

(22) $$\begin{array}{c} V\left(x_{ag},t\right)=\frac{1}{2}{\left(\dot{x}+\frac{1}{T}x\right)}^{T}L\left(\dot{x}+\frac{1}{T}x\right)+\frac{\beta }{T}\underset{i\in V}{\sum }\left|\xi_{i}\right|-\underset{i\in V}{\sum }\left({\dot{d}}_{i}+1/Td_{i}\right)\xi_{i}\text{.} \end{array}$$

and is absolutely continuous, over the set of values of *x*_*ag*_. Therefore, it is derivable almost everywhere, and its derivative is

(23) $$\begin{array}{c} \dot{V}\left(x_{ag},t\right)=-K_{p}\underset{i\in V}{\sum }{\left({\dot{\xi }}_{i}+1/T\xi_{i}\right)}^{2}-\beta /T^{2}\underset{i\in V}{\sum }sgn\left(\xi_{i}\right)\xi_{i}+1/T^{2}\underset{i\in V}{\sum }{\xi_{i}d}_{i}-\underset{i\in V}{\sum }\xi_{i}{\ddot{d}}_{i}\text{.} \end{array}$$

So, the system with a fixed network topology for connected and undirected graphs ensures that $\lim_{t⟶\infty }\;\xi \left(t\right)=0$ using distributed continuous control for appropriate control gain *β*. Thus, it is true that [54]

(24) $$\begin{array}{c} W_{1}\left(\xi_{ag}\right)\leq V\left(x_{ag},t\right)\leq W_{2}\left(\xi_{ag}\right)\text{,} \end{array}$$

where

(25) $$\begin{array}{c} W_{1}\left(\xi_{ag}\right)=\frac{1}{2}\lambda_{\min}\left(L_{0}\right){\left\|\dot{\xi }+\frac{1}{T}\xi \right\|}^{2}+\underset{i\in V}{\sum }\left|\xi_{i}\right|\left(\frac{\beta }{T}+\max_{i\in V}\left|{\dot{d}}_{i}+\frac{1}{T}d_{i}\right|\right)\text{,} \end{array}$$

and

(26) $$\begin{array}{c} W_{2}\left(\xi_{ag}\right)=\frac{1}{2}\lambda_{\max}\left(L_{0}\right){\left\|\dot{\xi }+\frac{1}{T}\xi \right\|}^{2}+\underset{i\in V}{\sum }\left|\xi_{i}\right|\left(\frac{\beta }{T}+\max_{i\in V}\left|{\dot{d}}_{i}+\frac{1}{T}d_{i}\right|\right)\text{,} \end{array}$$

*W*_1_ and *W*_2_ are positive definite and continuous, for $\xi_{ag}=\left(\xi ,\dot{\xi }\right)$ for suitable *β* which exceeds the term $\max\left|T{\dot{d}}_{i}+d_{i}\right|$. Moreover, it is true that [38, 43, 49]

(27) $$\begin{array}{c} \dot{V}\left(x_{ag},t\right)\leq^{a.e.}-W\left(\xi_{ag}\left(t\right)\right)\text{.} \end{array}$$

where

(28) $$\begin{array}{c} W\left(\xi_{ag}\right)=K_{p}\underset{i\in V}{\sum }{\left({\dot{\xi }}_{i}+1/T\xi_{i}\right)}^{2}+\underset{i\in V}{\sum }\left|\xi_{i}\right|\left(\beta /T^{2}-\max_{i\in V}\left|d_{i}/T^{2}-{\ddot{d}}_{i}\right|\right)\text{.} \end{array}$$

Eigenvalue decomposition of the Laplacian *ℒ* of the network graph yields

(29) $$\begin{array}{c} L=U\Sigma U^{T}\text{,} \end{array}$$

and using the equation *ξ*=*ℒx* holds

(30) $$\begin{array}{c} U\Sigma^{-1}U^{T}\xi =UU^{T}x=\left(I_{n}-\frac{1_{n}1_{n}^{T}}{n}\right)x=x-\frac{\sum_{i=1}^{n}x_{i}}{n}1_{n}\text{,} \end{array}$$

and consequently

(31) $$\begin{array}{c} x=U\Sigma^{-1}U^{T}\xi +\frac{\sum_{i=1}^{n}x_{i}}{n}1_{n}\text{.} \end{array}$$

Multiplying the above equality by (*e*_*i*_ − *e*_*j*_)^*T*^ results

(32) $$\begin{array}{c} x_{i}-x_{j}={\left(e_{i}-e_{j}\right)}^{T}U\Sigma^{-1}U^{T}\xi \text{,} \end{array}$$

and taking the limit at *t*⟶*∞* implies that $\lim_{t⟶\infty }\left(x_{i}\left(t\right)-x_{j}\left(t\right)\right)=0$ for every *i*, *j* ∈ *V*.

So, the collective price of the agents converges to the average price of their situations (average consensus).

Unfortunately, no other comparable model exists to serve as a benchmark. As a result, to prevent prejudice or false perceptions, we report the performance of the suggested model without comparing it to any other potential models.

## 5. Conclusion

In this paper, we suggested a preschool cyber security management system based on intelligent agents. We used cutting edge, intelligent techniques, and it aims to improve the ability of preschools to resist modern threats adequately, respond to cyber-attack incidents with the least possible impact, and protect their critical systems, services offered, and the personal data they hold and process.

The suggested system is a multiagent intelligent system comprising a group of agents that collaborate to solve the cyber security management challenge. The system intends to link and control distributed systems that currently exist, as well as to solve issues that are beyond the knowledge and skills of a single agent. Multiagent systems are a crucial area of distributed AI, where information is dispersed among distinct sources, such as previous experience in individual agent systems. The suggested multiagent network consists of a collection of agents with dynamic behavior collaborating to accomplish a common objective. The system in issue encompasses any network or system composed of geographically dispersed autonomous devices that collectively record circumstances and interact with each other through wireless or wired devices, exchanging data to provide an accurate estimate of the desired variable.

One of the core issues of multiagent coordination is the agreement, in which a group of agents must agree on a shared state value. The dynamics of the agreement protocol for undirected static networks are investigated in this work to implement the multiagent network of cyber security management.

Intelligent applications, such as the ones presented here, offer network defenders command over their environment, enabling them to turn the tables on even the most sophisticated attackers and stop them before they damage them. The need for interdisciplinary knowledge and deep experience in foundational cyber science skills such as understanding crypto analysis methods, building out security pipelines, and statistics, as well as computer science fundamentals and software engineering skills such as understanding computer architecture, proficiency with programming languages, and the ability to program software solutions is a significant disadvantage of multiagent applications.

Further research will be related to mechanisms of distributed control of intelligent agents in secure communications for other sector specific implementations. Also, I will be studying the multiagent reinforcement learning method that focuses on studying the behaviour of multiple learning agents that coexist in a shared environment.

## Data Availability

The data used in this study are available from the author upon request.
